# Exploring identity in coaching – insights into coaches’ understanding and approach

**DOI:** 10.3389/fpsyg.2024.1445643

**Published:** 2025-02-05

**Authors:** Alex Lazarus

**Affiliations:** Lazarus and Maverick Limited, London, United Kingdom

**Keywords:** identity, self, self-concept, identity coaching, identity work, coaching, executive coaching, whole-person coaching

## Abstract

Responding to a growing focus on identity within academia and the shifting socio-economic landscape, this study investigates the role of identity in executive coaching. Given identity’s complexity, its impact on human thriving, and its critical role in HRD, this research examines how coaches perceive, engage with, and address identity-related issues. Specifically, it explores how coaches navigate these issues without a standard theoretical framework, aiming to fill notable gaps in the literature on identity coaching and its methods. A qualitative, experiential approach was adopted, with purposive sampling used to select 14 experienced executive coaches from the United Kingdom, United States, and New Zealand. Data collection involved semi-structured interviews conducted over three weeks, followed by inductive thematic analysis, allowing key themes to emerge organically from participants’ insights. The analysis revealed five primary themes shaping coaches’ work with identity: the importance of a whole-person approach, identity’s central role in coaching, the coach as an instrument, methods and tools employed, and implications for coach education. While most coaches favoured an “inside-out” approach, emphasising the coachee’s self-concept and identity dynamics, they noted challenges in defining identity consistently, often shaped by their own experiences. Furthermore, while viewing this level of coaching as transformational, many also highlighted potential risks inherent in deep identity work, including unintended impacts on coachees’ personal lives. Ultimately, the findings underscore significant gaps in identity-related coaching frameworks and training, highlighting the need for more systematic approaches to addressing identity issues. To meet these challenges, the study recommends developing comprehensive resources and training to equip coaches for effective identity coaching practices.

## 1 Introduction

We all hope to feel good about who we are. In my coaching practice over the years, I’ve indeed observed that all human beings inherently strive for a positive self-concept, a fundamental component of human flourishing (Gecas, 1982). Yet, it is complex and influenced by a multitude of factors. It has been noted that historically identities have always been a powerful force, shaped by and shaping history itself (Harari, 2018; Burke and Stets, 2009), thus creating a dynamic, mutually constitutive relationship between the individual, societal and external changes.

In the context of today’s world, the Fourth Industrial Revolution continues to impact identities and alter social dynamics more rapidly than before creating unique challenges to all (Schwab, 2016). As individuals navigate these transformations, confronted with novel concepts, beliefs, actions and decisions, they may welcome these or perceive them as threatening to their identities. It’s also been suggested that these perceptions, if unaddressed, may negatively impact individuals’ sense of self, an outcome that should not be overlooked (Bostrom and Sandberg, 2011).

Recognizing that identity evolves in connection with others is key for coaches to be cognizant of this broader context and implications. For example, being more globally aware of each other than ever before (Hawken, 2009) connects the concept of identity with diversity as people explore who they are and who they are not (Villesèche et al., 2018) revealing endless choices of how to negotiate their self-image (Gergen, 2007; Ting-Toomey, 2015). As such, the notion of stable identity or identity as static achievement becomes an illusion (Stelter, 2009; Wetherell and Mohanty, 2010). Additionally, shifts in social status and power structures shed new light on how people perceive themselves and others (Future, 2001). As social dynamism increases, for some it equates to more autonomy in shaping their identities, free from the restrictive forces of earlier times (Future, 2001).

Another consideration is the changing employment landscape and how people approach their professional selves. With lifetime employment no longer a certainty, employability becomes a psychosocial construct and a cognitive compass that guides career identity (Fugate et al., 2004) powered by resilience, adaptability and ability to manage change proactively (Billett, 2007; Hall, 1996; Wu and Parker, 2011). Subsequently, these become key attributes sought by employers.

Rising life expectancy also opens a set of new possibilities and behaviors - living longer reshapes lives by challenging and changing people’s perception of age and fostering new habits and intergenerational relationships (Bostrom and Sandberg, 2011; Harper, 2013). It’s been suggested that it encourages flexibility in how individuals approach traditional life milestones such as education, work, and retirement, highlighting importance of cultivating agency and creativity for navigating diverse life paths in novel ways (Blatterer, 2007; Harper, 2013; Gratton and Scott, 2016; Gilleard and Higgs, 2016).

With these and other societal shifts like the climate crisis and geopolitical changes affecting daily life and threatening the future, coaching offers a way to help individuals process these realities. As a human being I’ve myself experienced those shifts and as a coach, I’ve frequently witnessed coachees sharing personal experiences connected to these broader issues and reflecting on their biographies in today’s changing world.

This connection between identity and coaching aligns with discourse that recognizes coaching as an answer to societal challenges (Stelter, 2016; Shoukry and Cox, 2018). Central to this perspective is the notion that the self is fluid and continually reshaped through introspection (Giddens, 1991), and the idea that links coaching with “soul healers” working with people’s inner selves (Western, 2017).

Building on this foundation, this research aims to deepen understanding of identity in the coaching process by examining how coaches interpret the concept of identity, how it shapes their practice and choices of methods and tools to help clients address identity-related issues.

## 2 Literature review

This section examines current literature focusing on the themes of identity and coaching, and the interplay between the two. It aims to locate helpful sources and identify gaps in the existing literature.

### 2.1 Identity – an open problematic

Although the search for what lies at the heart of who we are has fascinated scholars and philosophers for centuries (Stelter, 2018), it wasn’t until the mid-20th century that the term ‘identity’ entered the vocabulary of social sciences and humanities.

Furthermore, despite its centrality in various fields (Brown, 2015) and increased research across a range of theoretical frameworks, there’s no universally agreed-upon definition of identity. This may stem from new generations of scholars re-fashioning identity for the 21st century, revealing it as an increasingly complex, creative, and dynamically charged human experience (Wetherell and Mohanty, 2010). Identity also features prominently in research across multiple disciplines such as psychoanalytical theory, post-structuralism, post-feminism, and postmodernism, examined through the lens of morality, spirituality, gender, nationality and others (Wetherell and Mohanty, 2010; Leary and Tangney, 2011; Schwartz and Seth, 2011; Deaux and Snyder, 2012; McLean and Syed, 2015; Butler et al., 2017).

It is unsurprising that this multiplicity of perspectives leads to diversity of interpretations and ambiguities in academic research (Vignoles et al., 2011), resulting in fragmented, sometimes inconsistent or inaccurate terminologies (González, 2008; Zingsheim, 2011; Elliott, 2012; Ramarajan, 2014).

Indeed, identity remains an elusive concept - “an open problematic” (Wetherell and Mohanty, 2010, p. 1), “curiously puzzling and mysteriously contradictory” (Elliott, 2012) and any attempts to define it labeled “a wasteful detour” (Breakwell, 2015, p. 11).

Given the ambiguity surrounding identity, this study leans toward narrowing the framing of it, drawing inspiration from the Humanistic Movement of the 1960s, which influenced coaching psychology’s focus on self-realization, relationships, and phenomenological views of the self (Dryden and Palmer, 1997, as cited in Palmer and Whybrow, 2018; Stelter, 2013).

Therefore, the fundamental inquiry ‘Who are you?’ and ensuing answers reveal deep insights into people’s identity across various dimensions - personal, relational, and group - capturing values, beliefs, characteristics, relationships, roles and group affiliations. As such, identity acts as a meaning-making anchor guiding people’s choices, what they do and do not do, see and do not see, attend to and ignore, prioritize and avoid (Howard, 2004; Oyserman et al., 2012). While some put identity on par with other concepts such as character, self, self-concept and personality (Breakwell, 2015, p. 10), for the scope of this paper, terms of self, self-concept, identity, self-identity will be used interchangeably.

#### 2.1.1 Fixed or fluid? Given or gained?

Further exploration of identity concept reveals that, in the Western world, identity as we understand it today is a relatively recent construct. For centuries, it was largely determined by chance, assigned at birth and fixed within rigid social structures from cradle to grave.

By contrast, modern theories influenced by societal changes over the past centuries lean toward identity as a dynamic construct shaped by both individual experiences and societal changes (Fukuyama, 2018; Foucault, 1977 cited by Holstein and Gubrium, 2000). As noted by Fukuyama (2018), the rise of modern identity can be credited with the modernisation of societies a few hundred years ago and Foucault (1977 cited by Holstein and Gubrium, 2000).

Highlights that new jurisdiction and social sciences of that time transformed people from undifferentiated in a group to individual entities with social, legal and moral dimensions (James, 1892; Cooley, 1902; Mead, 1934). Thus, as social structures evolved, individuals sought new self-understandings and self-awareness, leading to emerging dynamics in identity (Snowden, 2010).

This reflects a broader understanding of identity as not merely a given but as continuously negotiated aspect of self through external forces and internal psychological dynamics. The shattering of identity construal as fixed was popularized by Freud’s work on identity as a process of psychodynamic, largely unconscious and inevitable psychological struggle within multifaceted psyche; id, ego and superego (Holt et al., 2012; Rowan, 2013). Though his work on early childhood identification was later challenged, his view of identity as fluid, contradictory, and partial (Frosh, 2010) inspired new insights into the two-way exchange between internal and external worlds (Hollway, 2010).

Where Erikson (Erikson, 1959) a few decades later advanced Freud’s theory by framing identity development to eight stages of life with tension and numerous identity crisis, Jung popularized the conflicting multi-tier view of people’s mind and their need for self-actualization and integration of the conscious and unconscious selves (Jung and Campbell, 1976; Snowden, 2006).

These progressive theories in sociology and psychotherapy initiated a shift from earlier notions of identity rooted in the Latin term *idem* and the French *identité*, implying consistency and permanence to identity as an evolving set of potentialities rather than a set of fixed traits (Rogers, 1961; Wetherell and Mohanty, 2010).

#### 2.1.2 Liberated or limited?

Another dynamic that may surface in coaching is the transformation of identity from a ‘given’ to a ‘task’ (Bauman, 2013). This shift reflects a growing need for individuals to engage actively in their identity work, balancing multiple roles and statuses (Erikson, as cited by Gleason, 1983) amplifying the importance of agency, self-direction, and choice (Schwartz et al., 2005).

However, while identity work is known to open scope for opportunities and flexibility, it can result in both creative or destructive process depending on people’s responses on how they navigate life (Leary and Tangney, 2011). Indeed, some argue that initial optimism over the democratization of societies encapsulated in the expression “chasing the American dream” (Zavalloni, 1993) ultimately produced a risk society (Beck, 1992).

In it, the saturated and reflexive self (Giddens, 1991; Holstein and Gubrium, 2000) has no choice but to make choices about their identities Beck (1992). Subsequently, the option of “do-it-yourself biography” (Gross, 1983 cited by Woodman, 2009, p. 245) can come at a price when the “freedom to become anybody” meant that nothing is ever over and everything always lied ahead (Bauman, 2013, p. 29).

Identity studies of the last century are particularly rich in insights into the tension borne out of the dangling promise of choice. On one hand lies the concept of a self-actualised, integrated individual (Rogers, 1961; Maslow, 1975; Jung and Campbell, 1976) blessed with the freedom of self-determination (Deci and Ryan, 1985, 2002). On the other, we confront the harsh realities of ‘fluid’ modernity, eroding traditions and decreased stability and security (Giddens, 1991; Beck, 1992; Sennett, 1998; Bauman, 1999, 2013; Stelter, 2018).

Stelter (2013) state that how people respond to these challenges hinges on their creativity and ability to adapt appropriately. While some may thrive in the “supermarket of identities” (Bauman, 2013), becoming self-made entrepreneurs of their identities (Walkerdine and Bansel, 2010: 494), others may struggle with the “tyranny of freedom” (Schwartz, 2001), constant evolving and becoming (Rogers, 1961) as if being perpetually “unfinished projects” (Bauman, 2013) and “provisional selves” (Wetherell, 2009). These individuals might falter, lacking the necessary psychological, social, and economic resources to keep pace (Stelter, 2018).

Subsequently, there’s an ongoing debate over the nature of identity (Black et al., 2017, p. 8), whether and to what degree it is fixed or fluid and influenced by structure or agency. Black et al. (2017), p. 8 conclude that weighting the balance of opinions for identity as fixed or fluid and the result of structure or agency continues to be of concern in identity theorizing. While Woodman (2009) acknowledges that identity in the Western world is increasingly seen as something to be personally constructed, he also warns against overemphasizing agency and neglecting structural constraints.

#### 2.1.3 Multiple identities

Another interesting aspect of identity is its multiplicity. The concept of multiple identities expanded upon by Giddens (1991, 2013) reflects the complexities brought forth by the VUCA world, which intensifies the need for a self-reflexive approach to identity that embraces multiplicity (James, 1892; Cooley, 1902; Mead, 1934; Giddens, 1991; Stryker and Burke, 2000; Gergen, 2007; Burke and Stets, 2009).

**Figure 1:**
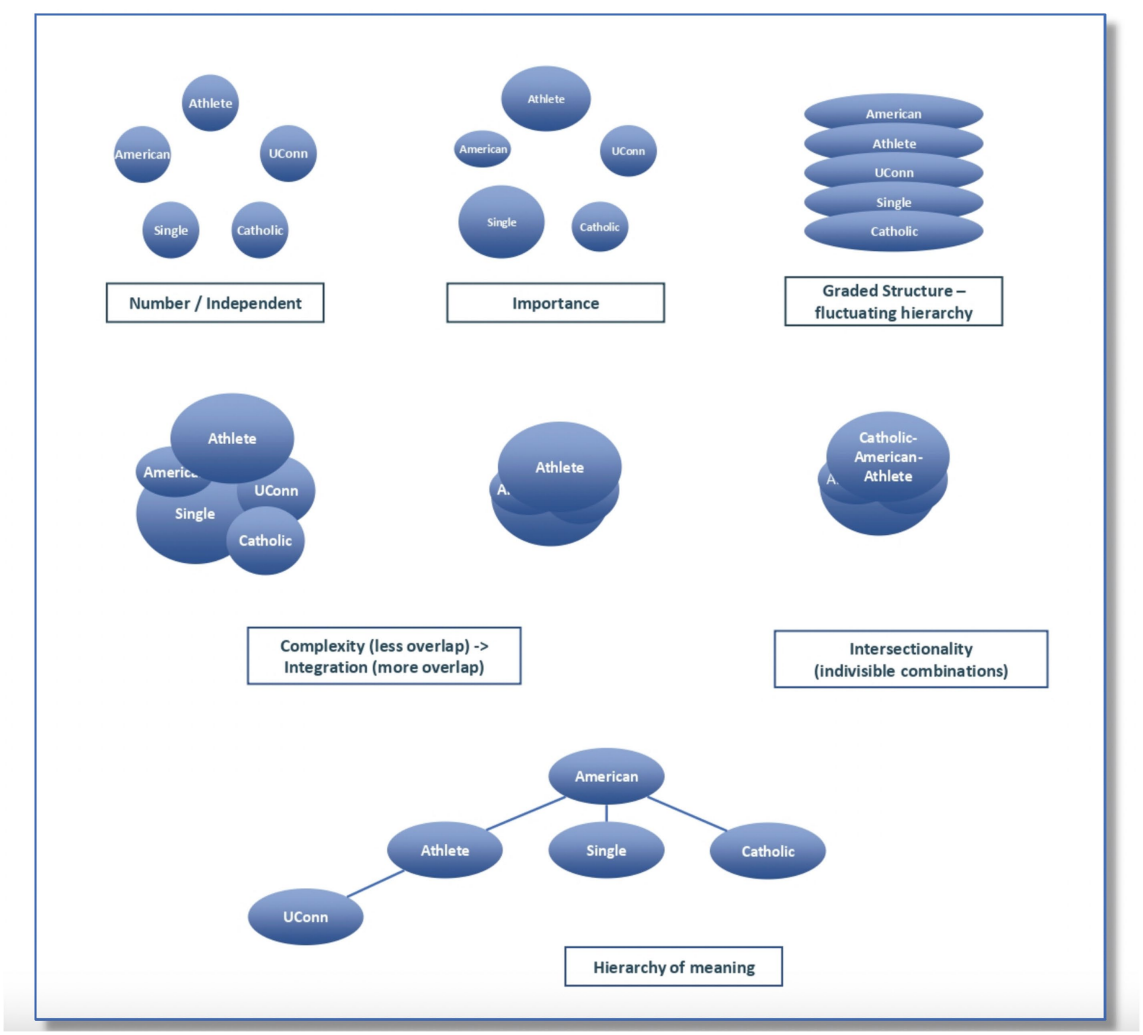
Illustrative images of multiple identities (Ramarajan, 2014).

While research in this area is still in infancy (Rowan, 2013; Ramarajan, 2014; Lester, 2017), it is understood that identities are situationally defined (Elliott, 2014) and people switch between their multiple identities based on perceived importance and relevance to the context in which they are activated (Burke and Stets, 2009; Rowan, 2013). Figure 1 demonstrates illustrative positioning of a person’s multiple selves.

Examples of how practitioners and scholars aimed to help people explore this concept over the years including methods for integrating or viewing different aspects of self as distinct resources, with constructs such as: the id, ego, and superego (Freud, 1923), the Child, Adult, Parent ego states in transactional analysis (Berne, 1968); complexes and archetypes in analytical psychology (Jung and Campbell, 1976), Gestalt’s topdog and underdog (Perls, 1992, p. 38), human psychology’s Theater of the Inside (Satir, 2009) and syndromes (Maslow, 1970); PCP’s community of self (Mair, 1977), self-complexity construct (Linville, 1985), the hoped-for-selves, possible selves and the feared selves (Markus and Nurius, 1986), characters (Bruner, 1990), subpersonalities (Rowan, 2013), subselves (Lester, 2017), forgone identities (Obodaru, 2017), mini-selves or self-models (Bachkirova, 2012) and many other variations.

**Figure 2:**
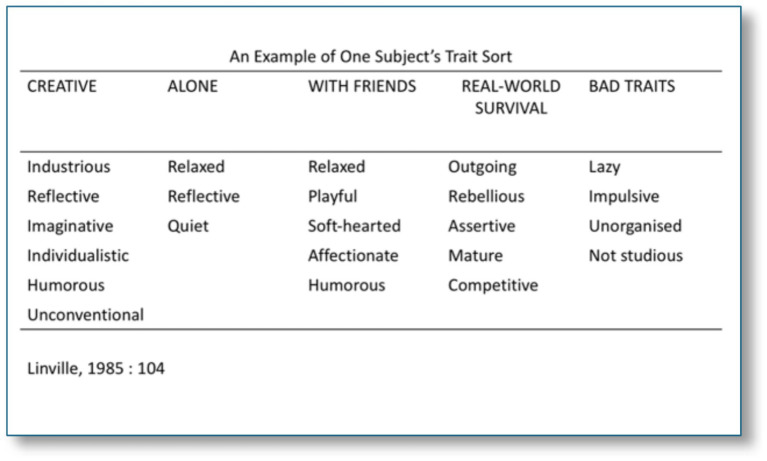
Self-complexity (Linville, 1985).

Some argue that having a collection of various selves or identities can provide numerous benefits. These include a wealth of resources, value, satisfaction, and an enriched life filled with diverse reference points and experiences (Sieber, 1974; Thoits, 1983; Linville, 1985; Thoits, 1986). Linville (1985), p. 94 notably describes how multiple selves can act as a buffer against the negative effects of stressful life events. An example from their research, outlined in Figure 2, details five sub-personalities of an individual and their varied responses to a particular aspect of self. Multiple selves are also considered beneficial for dealing with the contradictory aspects of human nature and its unrealised potential (Bruner, 1990), serving as a resource to improve adaptation (Nicolò et al., 2007), enhance flexibility, creativity, diversity, and versatility (Riha, 2011). Additionally, Caza et al. (2017) suggest that career pluralists can derive a sense of authenticity from multiple true selves, rather than behaviors tied to a singular true self.

Possible negative aspects arising from how individual’s process multiple selves can include contradictory aspects of self, unrealized potential, intrusive thoughts, self-talk, self-statements (Bruner, 1990:120). Some also suggest that the energy and effort required to manage multiple identities can lead to energy depletion, inner conflict, stress (Goode, 1960; Thoits, 1983; Rothbard, 2001), overwhelm and a sense of fragmentation of self (Gergen, 1999; Gergen, 2007).

#### 2.1.4 Identity as a lens for personal and professional development

Times of uncertainty and insecurity have been known to challenge personal identities, triggering identity work in individuals and formation of new identities (Woodward, 2004; Black et al., 2017). Similarly, it has been recognized that identity process bridges individual agency and self-creation with broader historical, cultural, and societal contexts (Edgar and Sedgwick, 1999; Watson, 2009). As such, in response to a world filled with challenges and constant change, Whitmore (2003) suggested that “You cannot fix the material world down there, you just have to develop yourself” calling for the “inside out” approach to tackling external chaos and working with the self to start with over controlling everything around us. Building on this, the World Development Report (2019) alludes to the role of identity on adaptability and self-efficacy, highlighting how these qualities are crucial for seizing both established and emerging economic opportunities, as seen in examples like protean (Hall, 1996), boundaryless, and nomadic careers (Cadin et al., 2000).

**Figure 3:**
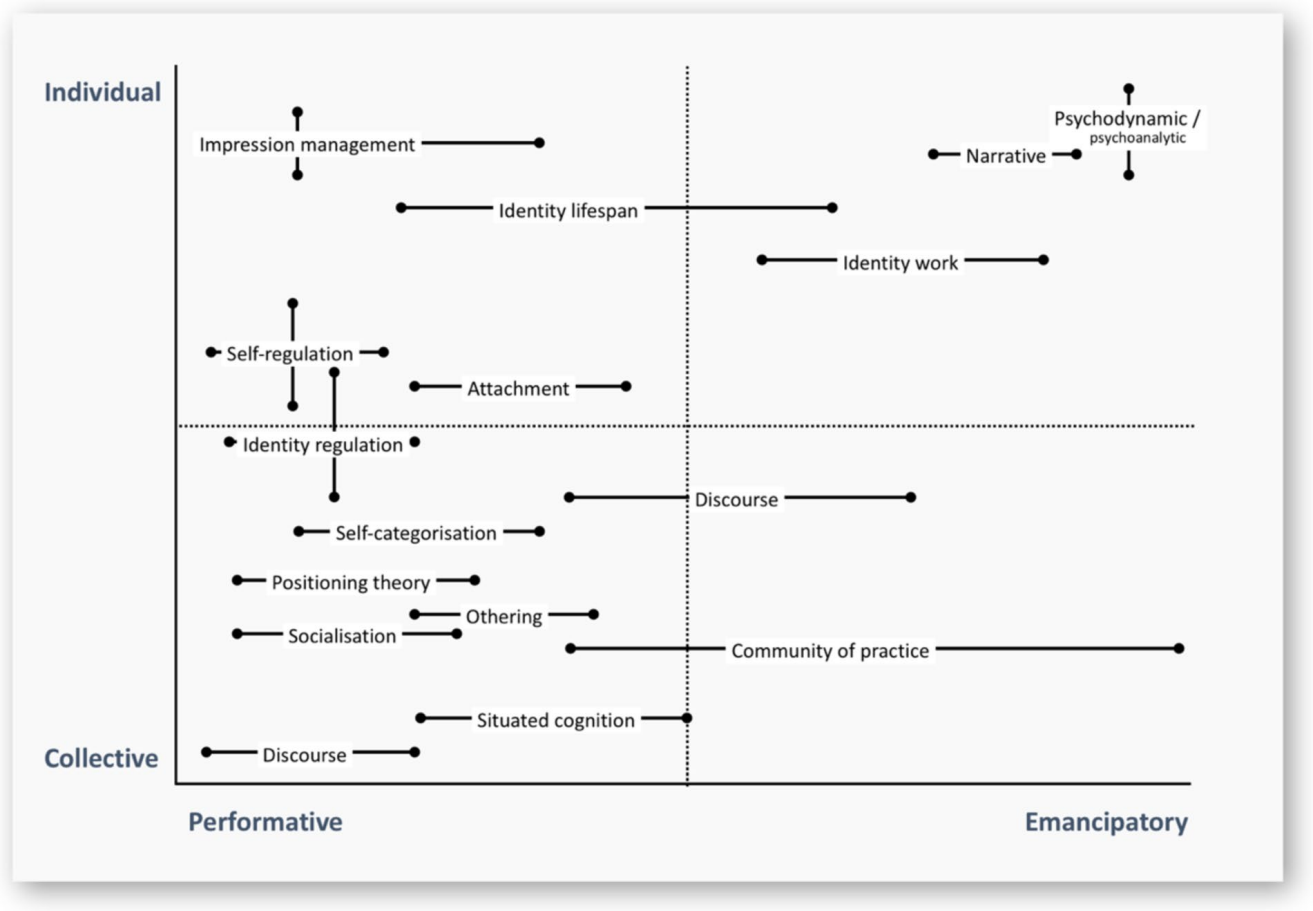
Typology of identity conceptual tools with potential for understanding HRD (Black et al., 2017, p. 12).

Subsequently, identity studies reveal profound impact of self-relevant constructions on individuals and wider organizations (Roberts and Creary, 2011). As a result, new forms of professional HRD practices have emerged integrating identity work into organizational development (Black et al., 2017). Furthermore, the identity-forming interventions in HRD demonstrated a high impact on both performative and emancipatory outcomes, boosting performance and agency, highlighted in Figure 3. Black, Warhurst et al. (2017), p.12 suggesting that identity and adaptability demonstrate higher competencies than basic skills and knowledge (Hall et al., 1997).

### 2.2 Coaching evolution - from instruction to identity

Having explored the complexities of identity, we now turn to the coaching literature, examining how it supports clients in navigating identity-related challenges and personal development.

Coaching has emerged as one of the fastest-growing services (Bresser and Wilson, 2010) and a pivotal element within Human Resource Development (HRD) (Egan and Hamlin, 2014). Much like psychology, counseling, and psychotherapy, some assert that coaching is increasingly viewed as an answer to late and postmodern challenges (Stelter, 2013; Shoukry and Cox, 2018). However, debates persist regarding the effectiveness of coaching in addressing contemporary challenges (Grant, 2017).

To better understand coaching’s current role, it is important to reflect on its roots, starting with first-generation coaching, which initially focused on goals and corrective performance enhancement, utilizing sports coaching principles of cognitive and behavioral modifications (Whitmore, 2003; Stelter, 2013). This approach was largely instructional, concentrating on control and correction with limited emphasis on self-reflection (Revans, 1982). It aimed to align individuals with organizational values rather than fostering freedom and creativity (McINNES et al., 2017). Consequently, it often failed to equip individuals for proactive and creative problem-solving (Binnewies et al., 2007) or address disruptions in self-understanding during organizational changes (Alvesson et al., 2008).

Second-generation coaching elevated the client to the expert role on him/herself in a process of deep listening and questioning. Although not without limitations and critique of its jargonised proprietary models (Grant, 2017), it was a positive move to draw more boldly from a humanistic perspective; Human Movement Potential; person-centered approach (Rogers, 1961) and adult development, thus emerging as “the art of facilitating performance, learning and development of another” (Downey, 2003), nurturing human growth and potential (Stober, 2006).

Building on this evolution, some have argued that for coaching to remain relevant, it must extend beyond individual performance and into the realms of human development and systemic thinking, seen as essential for helping individuals ‘connect the dots’ about themselves and others (Bachkirova and Kauffman, 2009). It recognizes individual’s efficiency is not merely an organizational issue but is deeply tied to personal attributes and psychology (Bush et al., 2013) and that individual’s sense of self as key for personal agency (Stelter, 2013; Billett, 2007).

Following this trajectory and responding to the growing complexity of human concerns, third-generation coaching has evolved into a partnering, relationship-based, morally complex activity (Hemmestad et al., 2010) addressing existential concerns about life’s purpose and meaning (Whitmore, 2003). Inspired by humanistic and increasingly spiritual perspectives, it facilitates exploration and development of a coherent identity, self-concept, and agency for a meaningful future (Whitmore, 2010; Bachkirova, 2012; Stelter, 2013; Stelter, 2018). It offers triple-loop learning, enhancing results, performance, thinking, and identity (Hargrove, 2008, pp. 114–116). All things considered, goal attainment will remain in the metanarrative of coaching, however, the significant shift is that the nature of goals will change and the road map of change is expected to be reversed (Bachkirova and Kauffman, 2009). Stelter (2018) agrees that coaching has to help people pay attention to one’s self-formation and reflective skills while working through goals and solutions at the same time. As such, the goal in coaching could indeed be understood as a product of identity expansion and broadening of horizons rather than a raison d’etre of coaching.

#### 2.2.1 The occurrence of identity in coaching

With identity being central to human functioning (McLean and Syed, 2015), it is expected for it to feature in the coaching agenda (Butcher, 2012). As coaching is said to be helping individuals reconnect with themselves (Whitmore, 2003), organizations seek coaching to address critical issues related to adult development, identity formation, and shifts in perspective (Fillery-Travis and Lane, 2014). Bush et al. (2013) agree that learning about ‘self’ through coaching impacts how people see themselves and the development of their identity. Others add that coaching can help clients narrate their future career identities and transitions (Black et al., 2017; Terblanche et al., 2018; Snape, 2021) assisting them in working through identity conflicts arising from the impact of social change and multicultural diverse society (Baron and Azizollah, 2014; Pinnock and Mayes, 2017).

However, given the pervasive concept of identity in human consciousness, the proliferation of coaching in the past few decades (Drake, 2008; Hawkins, 2013) and the recent emergence of identity in organisational studies (Bush et al., 2013), the literature review offers limited research on the specifics of identity coaching, identity construct in coaching and ways in which coaches deal with it (Snape, 2021). While studies referencing identity appear in various academic journals, few provide practical implications for integrating identity work into coaching practices, Bachkirova (2011) observes that when considering coaching of ‘the self’, one might have to ‘bite the bullet and try to take this challenge of creating a new approach to coaching’.

This yields a question of how well-equipped coaches are in this field? A 2017 survey exemplified this challenge, revealing that out of 82 coaches—90 percent of whom identified as executive or leadership coaches—54 percent felt inadequately equipped to handle identity issues. This highlights the need to establish a dedicated domain for identity coaching that complements existing cognitive, emotional, somatic, and spiritual methods (Pinnock and Mayes, 2017).

In the absence of theory on identity coaching, coaches might draw from diverse theories concerned with the self, including gestalt, NLP, existential, developmental, psychodynamic, transpersonal, ontological coaching, and more (Dilts and Bacon, 2007; Spinelli and Horner, 2008; Drake, 2010; Lee, 2010; Sieler, 2010; Stelter, 2013; Bluckert, 2014). They may turn to psychometric tools which add some, but limited, insight into personal identity (Passmore, 2012) or draw from a small number of studies with examples of arts-based, creative, narrative methods (Vähäsantanen et al., 2017; McGill et al., 2019; Snape, 2021; Vucic and Bolton, 2019; Steyn and Barnard, 2024). A similar gap exists in counseling and psychotherapy, where although identity is crucial, training seldom addresses how to cultivate ‘self’ as a reflective tool (Howard, 2004, p. 7).

#### 2.2.2 Identity coaching – competency

Can anyone coach on issues of identity? The attempt to answer this question must take into account that no agreed construct of identity in coaching exists and consequently, creating competency criteria might be a difficult task that leaves the question of ‘effective for what’ to be addressed first (Fillery-Travis and Lane, 2006). Notwithstanding the above, the literature review offers some initial insights worth considering.

##### 2.2.2.1 Basic assumptions

Hall (1996) states that knowing who one is, how to develop and how to extend one’s identity is essential to learning how to actually learn. However, he also stresses that adaptability and identity learning cannot be done alone. Wang and Millward (2014), p. 92 agree that ‘the coach should help the learner to build an identity from which the desired behavior is a natural response’ and De Vries et al. (2015) add that the more coaches understand how people’s selves are assembled, the more effective their coaching becomes.

However, for coaching to be successful, the coachee must feel comfortable to reveal all the various parts of their self to the coach (Pinnock and Mayes, 2017). Bush et al. (2013) also argue that while it may be a challenge for coaches, they must have both the understanding of the coachees’ identity and the aptitude to enable them to explore their personal, group and cultural identities in a way that helps them adapt to changes within their environment.

Flaherty (2006), p. 19 goes even further by stating that unless coaches can reveal to themselves what they understand human beings to be, they should not coach and add that “without this understanding, it’s as if we are attempting to build a structure with materials that we aren’t familiar with.”

##### 2.2.2.2 Professional mastery

Fillery-Travis and Lane (2014) argue that those who work with coachees’ fundamental belief systems and help clients explore intimate, professional and personal questions must demonstrate highest levels of professional mastery. The authors express concerns over coaches who engage in psychologically-based concepts without an adequate understanding of psychology. Equally, Berglas (2002) highlights possible dangers and limitation when coaches who only operate on the behavioral level, fail to understand how to deal with client’s intrapsychic pressures which often create ineffective behaviors in the first place.

Taking the constructivist lens of identity as a broad, unexpected and an open topic, Du Toit (2007), pp. 118–136 stresses that coaches should be able to flexibly respond with different skillset to the different needs of different clients (Du Toit, 2007, p. 136 citing Stewart et al., 2008) and operate at system eclectic level which requires time and coach maturity through an immense amount of reflection, experimentation and adaption of practice (Clutterbuck and Megginson, 2011). Adding to the above, Bachkirova (2012) proposes that coaches involved in developmental work ought to be mindful of their own growth and ego, and that such work may better suit a well-trained external coach rather than a manager or an internal coach.

##### 2.2.2.3 Thin line between coaching and therapy

As noted, issues of identity benefit from coaching psychology which offers many of its models grounded in therapeutic approaches (Grant and Palmer, 2002) and the aspect of facilitating personal change does not set coaching apart from its other sister practices (Hawkins, 2013, p. 747). Developmental coaching, in particular, which has been credited with creating a personal reflective space has also been likened to “therapy for the people who do not need therapy” (Fillery-Travis and Lane, 2014). While coaching is indeed often therapeutic, it is not a substitute for therapy and professional coaches have to differentiate between clinical and non-clinical issues (Grant and Cavanagh, 2004). Thus, to prevent coaching stepping over the fine line toward counseling, it is important for coaches to have the self-discipline to recontract and stop when the coaching agenda enters an area of their insufficient skill or goes deeper than what coaching was contracted to help with in the first place (Whitmore, 2003; Bachkirova and Kauffman, 2009).

##### 2.2.2.4 Evidence-based coaching practice

Grant and Cavanagh (2004) observe an increasing interest for coaches to ground their practice in a solid theoretical understanding and empirically tested models. Drake (2008) agrees that coaches benefit from a deeper understanding of applied sciences that underpin coaching (Drake, 2008). Pertinent to this is Schwartz’s (2001) argument that understanding the structure of identity is of paramount importance if theorists and researchers are to study it and work in a capacity that promotes its development. Subsequently, coaches wishing to or currently coaching on issues related to identity ought to reflect on their level of knowledge in this area.

The literature review recognized the rising importance of identity as a theme in HRD and coaching, and it reviewed a number of perspectives related to identity as a concept which coaches might encounter in their work. Where the literature has been most illuminating was in revealing the complexity of identity as a fundamental human experience. However, the literature review also captured a lack of theory of identity related specifically to coaching and limited and understanding of how coaches work with this complex phenomenon.

## 3 Materials and methods

The research objective was to explore coaches’ knowledge, awareness and understanding of identity in the process of coaching, and their choice of methodology and tools when coaching on issues of identity.

As stated by Willig (2013), research design should be influenced by the type of knowledge the researcher wished to produce. In this research, since identity is a difficult construct to measure (Schwartz, 2001) and there’s no common theory that binds identity and coaching (Butcher, 2012), the best solution was to position executive coaches as a direct source of data. Subsequently, their contribution, insights and interpretation related to issues of identity in coaching were of key importance.

### 3.1 Philosophical orientation

Since the research hopes to generate insights directly from coaches, as a researcher I dismissed the notion of unitary reality or objective knowledge assumed by positivism (Willig, 2013, p. 7). Rather, the underlying ontological idealist position adopted by the paper assumes that reality is dependent on people’s mental structure and activity (Guba and Lincoln, 1994 cited by Slevitch, 2011) from which they construct an “outer” reality (Smith, 1983, p. 8). As such, I accepted that coaches’ views were subjective aligned with philosophical interpretivist stance that people decode the world around them in a particular context (Rose et al., 2014) and which is more concerned with emphatic understanding than explaining the human experience, behavior and action (Bryman, 2016). Inviting coaches to share insights and observations acknowledged that knowledge does not only arise from experience but also from reflecting on (Ormston et al., 2014).

### 3.2 Experiential qualitative research

For research methods, I reviewed the benefits of a quantitative approach for its potential to generate broad data. The breadth, however, might have restricted an exploration of an emerging field by focusing on predictions, counting occurrences, volumes or the size of concepts known to this approach (Smith, 2015). Furthermore, I was concerned about my subjective preconceptions seeping into the design in the absence of solid findings in the literature review.

Subsequently, driven by qualitative focus on experience, understanding, perceptions, views and opinions, I chose inductive experiential qualitative approach known to offer a way to understand participant-defined meanings, how they experience and manage situations related to the research question (Willig, 2013) thereby building theory with the help of their complex accounts (Braun and Clarke, 2006).

Mixed methods were considered for this study, but ultimately rejected as they did not offer the best approach for answering the research question. Factors such as time constraints, challenges in integrating data from differing epistemological assumptions (Lyons and Coyle, 2016), and my level of experience as a researcher led to concerns that using mixed methods could jeopardize the overall quality of the research.

### 3.3 Sampling strategy and sample size

**Figure 4:**
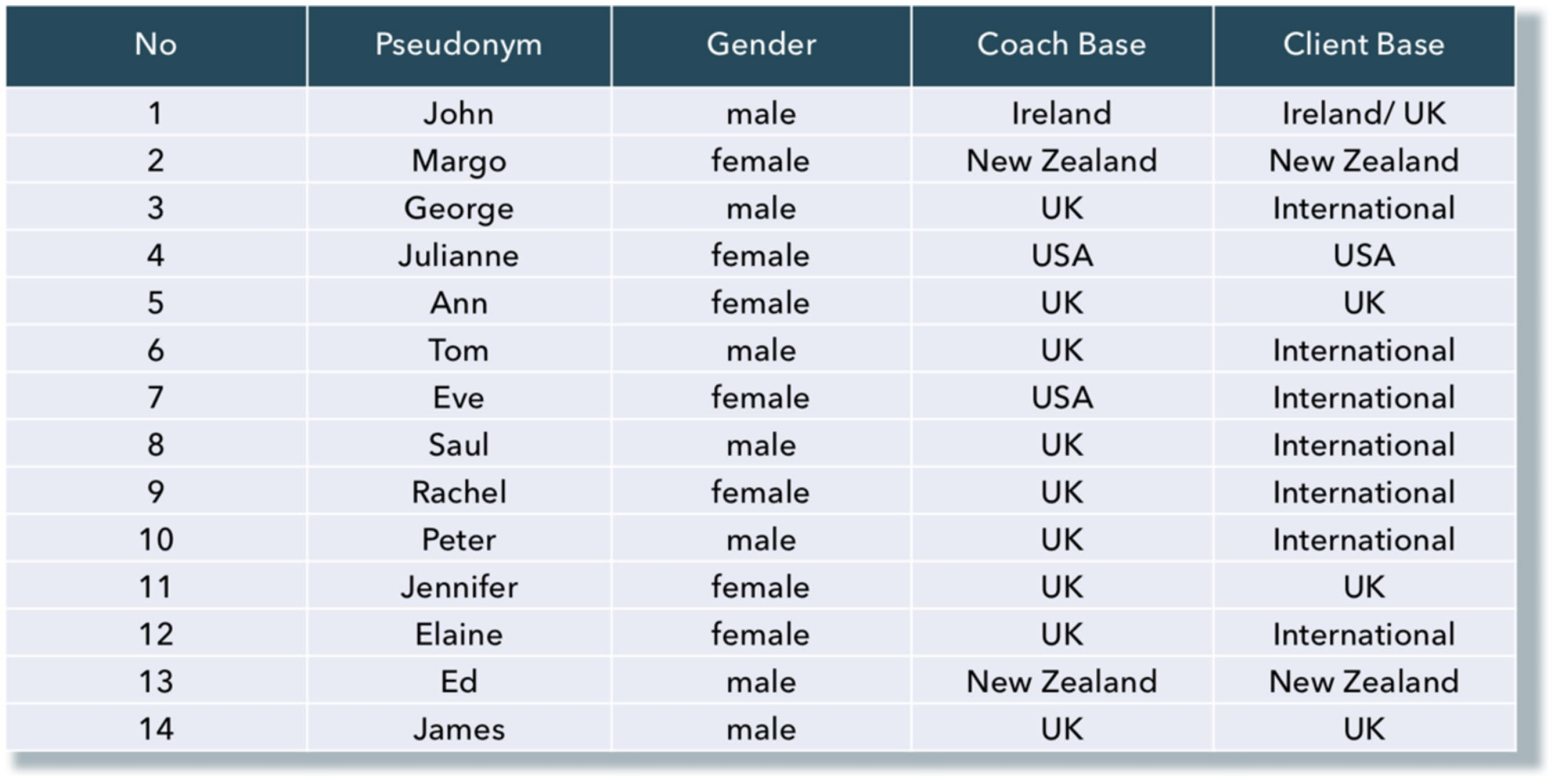
Research sample.

Participants were selected using a purposive sampling strategy, chosen for its appropriateness in meeting key criteria relevant to the subject matter (Ritchie et al., 2013). This approach ensured the selection of individuals who could provide ‘insight and in-depth understanding’ related to the research question (Patton, 2002). To maintain homogeneity, all participants were executive coaches actively providing services. The group consisted of 12 external coaches, one internal coach, and one with a hybrid role. To ensure a diversity of perspectives and account for the impact of different characteristics (Ritchie et al., 2013), the sample included an equal number of male and female coaches, with varying levels of professional tenure, education, and experience. Participants were based in the United Kingdom, United States, and New Zealand, and all were experienced in working within diverse multicultural contexts, as shown in Figure 4.

### 3.4 Time horizon and data collection

The research adopted a cross-sectional approach, as there was no need to assign chronological sequencing to events to produce the desired data (Rose et al., 2014). Consequently, data was collected through non-sequential interviews with coaches, conducted within a three-week, time-bound period.

Following Creswell (1998) observation that qualitative researchers build complex, holistic pictures by analyzing participants’ words in natural settings, I familiarized myself with various data collection methods, including diaries, journaling, and ethnographic approaches.

However, semi-structured interviews lasting between one and 2 h were considered optimal for obtaining data and meeting the purpose of the research described by Rose et al. (2014), p. 4 as a systematic process of investigation aimed at finding solutions to a problem.

In addition to practical consideration such as research project management time frame and access to participants, the semi-structured interviews are known to be a great way to collect meaningful data capturing meanings and descriptions relevant to the research topic (Kvale and Brinkmann, 2009). Given the absence of a theory to be tested, an inductive approach was adopted, allowing for the identification of themes and patterns to build an emerging theory based on observations (Rose et al., 2014).

The pilot interview and subsequent reflection provided valuable insight into managing time effectively and balancing the need to keep the conversation relevant to the research topic while allowing coaches the freedom to explore and generate unanticipated insights.

### 3.5 Interview guiding questions

Guided by Braun and Clarke (2006), p. 78. I used a set of pre-written interview guide questions (Appendix 1), while allowing participants the flexibility to raise issues I have not anticipated.

For the purpose of this research, I assumed the role of an interviewer seeking direct input from coaches. The interview questions were designed to align with the key areas of exploration, as detailed in Appendix 1. While I remained open to unanticipated insights, the questions served as prompts when the conversation veered off-topic. Over time, I simplified the wording of the questions and replaced the word “issue” with “theme” to avoid suggesting that the research was solely focused on problematic aspects of identity coaching.

All interviews were conducted virtually and recorded using an audiovisual meeting platform. Two interviews were transcribed verbatim by an external transcription agency, while the remaining 12 transcribed using a software program. The software’s function of reading along with the voice recording, with the option of simultaneous data mapping, allowed me to recreate the interview experience by replaying it multiple times, enabling me to become intimately familiar with the dataset. This was significant because transcripts are generally ‘two-steps removed from the actual interview experience, and with each step, information is lost or changed in some way’ (Braun and Clarke, 2006: 162). Finding this process both intuitive and effective for working with the data, reducing it, and applying codes relevant to the research question, I decided to redo the first externally produced transcripts using the same method.

Printout of the transcripts with initial codes applied digitally were then taken through manual coding phase, working line by line, highlighting appropriate segments of data and noting the same code in the margin (Rose et al., 2014, p. 342). This allowed me to interact with the data differently, reading the words actively, analytically, and critically (Braun and Clarke, 2006). In parallel, I documented my thoughts in a journal from the moment the first transcript arrived, making the data analysis a month-long process of immersion and reflection.

### 3.6 Data analysis

Thematic coding is widely used across qualitative methods in the social sciences (Braun and Clarke, 2006, p. 174) and was chosen for this research due to its theoretically flexible approach, which is fitting given the lack of established theory in identity and the focus on a novel idea. It guided me through the process of identifying, analyzing, and reporting patterns or themes across the data in a structured and systematic way. As explained earlier, the digital transcription software was invaluable in identifying codes, patterns, and building themes over multiple stages during a three-week period. From the first interview, I began noting items of potential interest (Braun and Clarke, 2006), expanding the analysis with each transcription as it became available.

### 3.7 Ethics and limitation

The research participants were first contacted via introductory emails, and when possible, in person. Formal invitations followed, outlining the research objective, rationale, interview process, and permissions. Upon receipt, these documents were securely stored, and key ethical considerations were reiterated at the start of each interview to ensure all participant questions and concerns were addressed.

Throughout the research process, I remained mindful of both the limitations and strengths of qualitative research, particularly the close proximity of the researcher to the participants and the influence of my own viewpoints. A key learning point was recognizing the axiology of interpretivism, which asserts that research is value-bound, and the researcher’s subjectivity is reflected in the observations made (Rose et al., 2014). This raised early questions about what constitutes good research, for which Guba (1981) criteria for trustworthiness and authenticity, credibility, dependability, confirmability, and transferability served as a valuable guide. Consequently, I remained aware of my dual role as both a researcher and an executive coach, facilitating change in organizations. Like the participants, I was also creating meaning through my thinking and interaction during interviews (Kivunja and Kuyini, 2017). This recognition, as described by Braun and Clarke (2006), p. 207, positioned me more as a sculptor shaping the content of the interviews, rather than an archeologist simply uncovering it (Kvale and Brinkmann, 2009).

Given that ‘no straightforward tests can be applied for reliability and validity in qualitative research (Patton, 2002), I adopted a practice of self-reflexivity, focusing on my mindset, intention, and expectations before, during, and after each interview (Schon and DeSanctis, 1986). Journaling and iterative data evaluation also helped surface unhelpful assumptions, which I subsequently challenged.

## 4 Results

**Figure 5:**
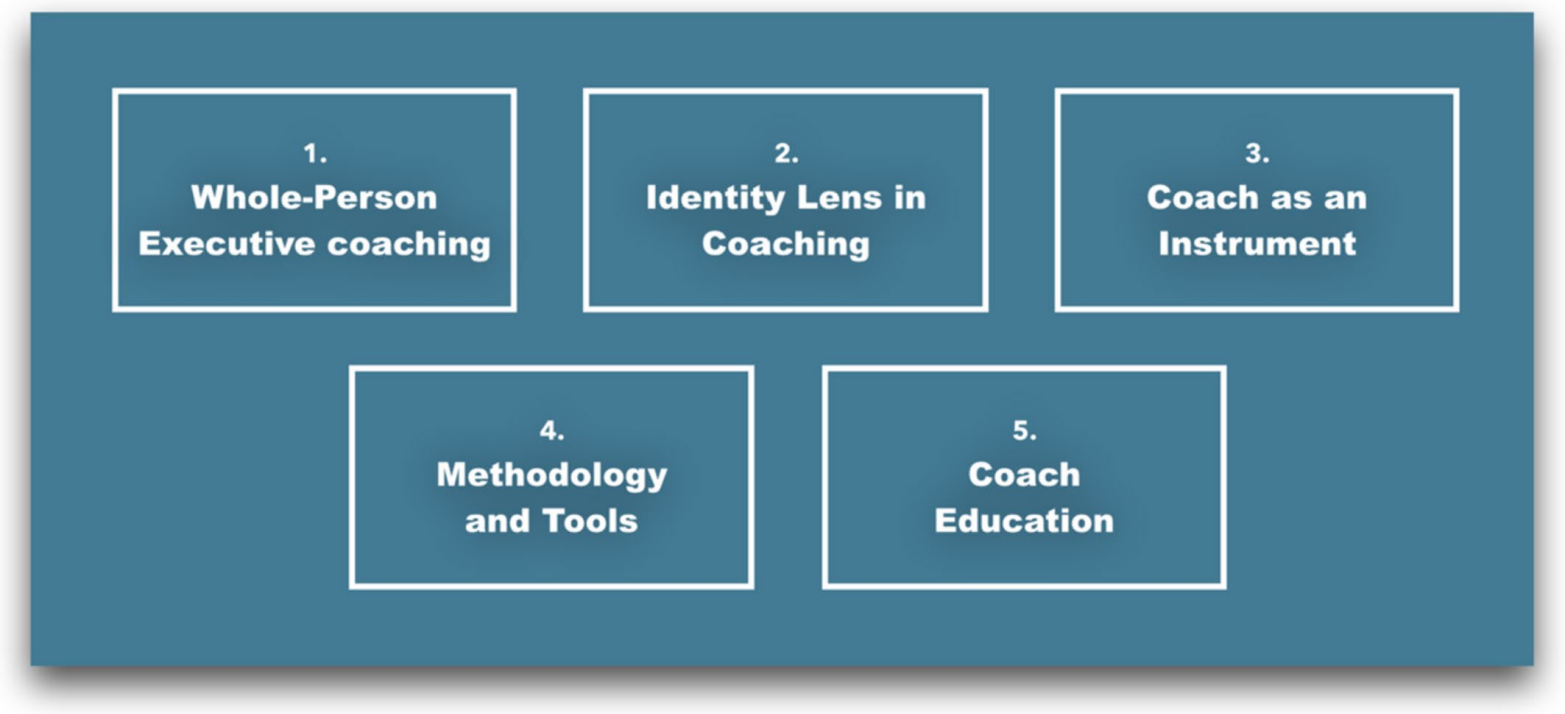
High level themes (Lazarus, 2019).

The research revealed recurring patterns in the data through comprehensive thematic analysis. These patterns were organized into five themes, presented in Figure 5. This classification aligned with research objectives and provided crucial insights for those seeking to enhance their awareness, understanding and practice of identity coaching.

### 4.1 Theme 1 - whole-person executive coaching

#### 4.1.1 Beyond goals: exploring whole-person development in coaching

The interviews revealed a transformative shift in executive coaching, evolving from a primarily goal-oriented, skills-focused approach to a richer, more dialogical process that explores the depths of personal essence rather than just addressing actions and goals. This was perceived as a deeply nurturing journey that not only enhanced the well-being of coachees but also fostered sustainable growth beyond mere task achievement. This was prevalent among independent coaches who tended to lean toward whole-person coaching, fostering personal transformations and underscoring a shift toward valuing holistic personal development. Slight variation to this came from a perspective of an internal coach who deployed more traditional focus on aligning coaching with strategic business goals, especially during periods of transitions. In terms of topics generally brought to coaching, in addition to typical themes of leadership development, promotions, and team-building and others, the scope has significantly expanded to address the full spectrum of an individual’s life, including cognitive, emotional, behavioral, physiological, and occasionally spiritual dimensions.

This holistic approach has also revealed how coaches perceived their role and contribution focusing on outcomes that cultivate a deeper relationship with oneself and others. This shift emphasized not just achieving specific goals but rather how these relationships impacted goal attainment. While it was agreed that solution-seeking would remain a core component of coaching, majority of coaches preferred to address emergent issues as captured by one of the coaches, Tom: “*Thinking about it, I rarely set goals at the outset. Just let those emerge and then maybe summarize some back.”* Similarly, Elaine, a coach with extensive coaching experience, viewed working with goals as more superficial: *“(...) obviously there are coaches who just work at the surface level in terms of, you know, what’s your goal? What are your options? Here’s way forward, but it’s not the work I tend to do.”*

Reflecting on core aspects of his coaching, James positioned self-awareness as a dominant theme: *“The self-awareness piece, which obviously is core to our work and coaching and helping them be aware that it’s important that they take note of how they feel and what’s around them and take time to do it, to reflect, reflect, reflect.”* Another coach, Ann, asked about her coaching focus, identified authenticity as: *“(…) being that person all of the time. You know, all aspects of one’s life.”* The person-centred approach to coaching was also illustrated by Peter, a coach who works with successful executives in commercial settings: *“My coaching philosophy is to help people see the nature of how they are coming across, but also how that’s being created through thought, how they operate in the world, through their mind. In the context of this, you might say, through their identity because it seems to me that we do not show up in the world through some kind of objective sense.”*

Recognizing the daily challenges her clients face, Rachel’s humanistic approach to coaching became evident when asked what clients needed from coaching the most: *“(…) sounding board but also to be vulnerable and people who aren’t going to judge you for saying, ‘I do not know what to do next, I’m confused, I’m scared, I do not know who I am.”*

#### 4.1.2 Not therapy, but therapeutic

Regardless of whether coaching served high performers or remedial intervention, many coaches recognized their clients’ struggles with action bias, confidence issues, work-life imbalance, emotional deregulation, and burnout. It was also noted that clients were becoming comfortable with discussing their mental health with coaches, with some coaches’ proactive efforts to prepare for this conversations. Rachel, for example, selected coaching training programs that incorporated therapeutic techniques and Adam shared that he purposefully integrated personal coaching for professionals interested in wellbeing and optimal functioning. Similarly, Julianne, a coach with over 12 years of experience as a certified executive coach, has recently completed a life coaching course and stated: *“I take the whole person approach to coaching (…) I am an executive coach who infuses some life coaching into my executive coaching.”*

Given the complex backdrop of work in the 21st century, it’s unsurprising that questions about the philosophy and ethics of coaching prompted reflection and a realization of the type of coaching they practice. While many have been mindful not to conflate coaching with counseling, on reflection some agreed that their coaching was producing some therapeutic value.

This can be illustrated by James, a former board member of a well-known global organization, and now a coach in the business coaching domain who observed: *“Very often CEOs are people who are themselves deeply troubled and unhappy and the energy that they have given to the business that they lead is so all consuming that it’s taken a lot more out of them that they are able to give. So we have this notion of kindness for CEOs and that’s generosity of spirit.”* John, an executive coach with background in leading commercial teams, was candid about his principles grounded in empathy and shift in self-related wellbeing as he emphasized the importance of authentic self-acceptance in his coaching practice: *“I’m helping people to see that they are okay (…) So, that’s kind of where I am in coaching, maybe it’s controversial (…), this idea of coming home to yourself.”* Aligned with this approach, Saul added: *“I’ve always found that psycho-education and self-understanding and self- awareness provide an avenue and route to self-acceptance.”*

Reflecting on the impact of coaching, which aims to expand people’s understanding of their own behaviors and facilitates moments of new insight, coaches agreed their clients found liberating to view their often complex and private situations from a new perspective.

This section recognized that executive coaching is becoming enriched and nuanced with human-centered, whole-person compassionate component, encapsulated by a quote by Saul, a former management consultant-turned-coach who cited it as inspiration for his coaching: “*The human spirit steps forward when people are held lovingly and firmly to the standards of which they are capable.’ You asked me what I want my coaching to be about or to grow? That’s it”*.

### 4.2 Theme 2 - identity lens in coaching

#### 4.2.1 Defining identity

Despite a preference for transformational coaching, most coaches were unsure of how to define identity, using expressions indicating exploration rather than confirmation such as: *“I guess,” “I take it for granted,” “not sure,” “never thought about it before,” “I could be wrong,”* and *“oh dear, there’s a challenging question”*.

Identity as a concept presented itself as elusive and difficult to articulate and emerged first from personal and professional experiences rather than theory. Most coaches reflected on their own identities and their identity as coaches first before trying to build the concept of identity. In absence of one theoretical source, some coaches drew from psychological frameworks such as adult development theories, transactional analysis, psychodynamic theory, and Neuro-Linguistic Programming (NLP).

In shaping their definition, coaches referred to storytelling, Buddhist principles, neuroscience, systems thinking, and others forming an eclectic mix of principles expressed in their coaching. Peter articulated his understanding of identity by describing it as having both an internal and external aspect. He explained: *“(…) It’s an inward dimension and an outward dimension. It’s the conversation that you have with yourself about what am I displaying to the world, what I am feeling and thinking and doing, and how is that conversation working for me?.”* Margo likened identity to a wardrobe, suggesting it offers a range of behavioral choices and personas depending on the context, thereby illustrating identity’s adaptability: *“I used to think that identity was this finite kind of a core who you are, but the more I do this work, the more I think that identity is almost like a wardrobe, full of identities or different kinds of personas”*.

Rachel added: *“I would say it’s like religion. There are many doors to the same room. And they have different colors. Knock on each door and walk through it and you’ll get closer to the answer. You will not get the answer. You’ll get closer to it”*.

The point of commonality was that identity is a relational dimension with self, other people and the system in which people exist and influence, and some recognized that there are many sub-identities. There was no agreement either whether the concept of multiple identities was beneficial or a hindrance, as contemplated by Ann: *“Maybe you can have multiple identities that remain authentic? I’m making it sound like you can only have one identity. But maybe, it’s okay to have more than one? There’s a question. Maybe it’s appropriate? Now I’m talking to you about it, maybe I’ve got multiple identities?”*

All coaches agreed that identity forms the fundamental, intrinsic part of oneself that shapes values and guides behaviors, and all coaches acknowledged spiritual and physical aspects on one’s identity, adding layers of complexity and richness to identity definitions. While elaborating on their interpretation, many acknowledged that drawing specifically on their preferred coaching modality as influencing factor for defining identity held both positive as well as prescriptive, and therefore a limiting, impact on their coaching around identity.

#### 4.2.2 Identity in coaching

While majority of coaches preferred transformational coaching, it was evident that those with stronger psychological background were quicker to assert that all coaching links with identity as exemplified by Rachel’s reflection: *“I’ve never thought about it—if you are not working with identity, what are you working with? It always goes back to challenging the self and challenging identity in a supportive way and offering different perspectives.”*

Elaine added that since all behaviors reflect one’s identity, this necessitates a thorough exploration for meaningful change: *“I think that deeper level is really important for people to explore because for me in coaching that sort of transformational change happens here rather than surface level change. I think if people make changes at that level, it’s more likely to be sustainable”*.

Reflecting on identity coaching in his practice, James agreed: *“In my 12 years of coaching, those individuals with whom I’ve achieved the most lasting change are those who delve deeper into discussions about identity”*.

One of the more prominent insights of identity as practiced in coaching was the sense that it linked to something deep, as noticed by Margo: *“You know, you are delving into the deep psychology of my essence, what drives my beliefs and assumptions—it’s quite profound”*.

Interestingly, this phenomenon of ‘depth’ emerged repeatedly during the interviews, manifesting as a significant element of identity coaching. Some coaches were naturally drawn to the concept of depth and even experienced frustration when their coachees appeared reserved and cautious. Rachel shared: *“When things feel too good to be true and you constantly encounter only the shiny surface.”* When the opposite was true for Tom, he expressed satisfaction and fulfillment, when exploring the deeper layers of self with his coachee: *“When they are willing to share, for me that’s where I actually almost get excited”*.

However, as interviews progressed with discussion on depth, some coaches started to approach the concept of depth with caution, viewing them as potential red flags indicating areas that might be inappropriate to probe. ‘Depth’ was interpreted in various ways, often serving as a measure for coaches to re-calculate their professional boundary. While everyone acknowledged that identity encompasses a person’s past, the main point of dissonance and discomfort circled around past experiences and, especially, in childhood and early adolescence. As noted earlier, the pattern noticeable in the data was that coaches with stronger psychological orientation were more likely to explore coachees’ past to work with their ineffective behaviors whereas others were more cautious and asserted that anything grounded in the past belonged to psychotherapy (Lazarus, 2019). James, bearing his clients in mind, categorically signposts his boundary on this topic: *“For something that occurred to them in the past but affecting their business performance, they can go and get sorted out in their own way with a psychotherapist”*.

When asked about the recurrent themes associated with identity in sessions, coaches cited issues of self-belief, self-esteem, outdated perceptions of self, confidence, internal conflicts, achievement focus, perfectionism, imposter syndrome, lack of direction, a perceived loss of control, feeling overwhelmed and anxious, relationships, interpersonal conflicts, personal branding, career advancement, retirement, parenthood, grief, relocation, gender, ethnicity, immigration.

Coaches were not all in agreement a to the true impact of coaching around those issues. More psychologically inclined coaches, as observed earlier, had more confidence in coaching being the right intervention while others like James disagreed expressing his perceived limitation for coaching: “*I do come across perfectionism. I think what’s interesting about this is, as you know, these are things which are quite difficult to shift as executive coaches as opposed to therapy or counseling”*.

Reflecting further on the concept of identity in coaching revealed that most coaches have not fully considered their identities and the distinction between personal and coach identity. Subsequently, on reflection, some coaches deemed important for people to develop a coherent identity by thoughtfully and effectively integrating various facets of the self to alleviate internal conflicts, reduce stress and enhance agency, choice and flexibility to adjust self-concept. Reflecting on this, Margo, specializing in leadership development, noted: *“People aren’t aware of the identity they have been given by others. Helping them to identify that can help them be aware of ways forward (…) to lead more autonomous, more fulfilling lives.”* Adding to this point, George said: *“I think the greatest opportunities is to understand and really embrace the idea of emergent identity, to look at our background and our history and our story with fresh eyes and decide what we want to take forward and what we want to leave behind because it’s no longer helpful”*.

Ultimately, most participants agreed that identity coaching is instrumental in swiftly addressing maladaptive behaviors, empowering individuals to effectively plan and envisage their futures with readiness and enthusiasm for impending challenges or goals. Beyond personal gains, coaches observed multitude of outcomes beneficial in an organizational context including fostering authentic and adaptive leadership, strengthening team cohesion, enhancing collaboration and innovation and facilitating the swift transition into new organizational roles.

As outlined above, identity-based coaching can deliver substantial benefits to coachees. However, it is crucial to consider the potential risks associated with delving too deeply into personal identities. Notably, coaches who initially saw minimal risk in delving deeply into aspects of identity revised their opinions during discussions. Upon reflection, many were surprised and became concerned about their initial oversight of potential risks. Majority agreed there were risks ranging from mild to significant, and damaging to the coachees, and some acknowledged their insufficient awareness in this area, as demonstrated by a comment by Ann: *“I’m not sure that most of us when we are coaching actually identify with a risk when we are working at identity level”*.

Reflecting on risk, it was broadly agreed that addressing identity-related issues could make coachees feel vulnerable, exposed, and even intimidated. Interestingly, some discomfort seemed to arise when coachees were encouraged to critically examine their own identities and the meanings they attached to them.

There was also a concern about the irreversible unintended consequences of identity coaching – through a-ha moments, temporary elation, clouded judgment, inflated potential, or event expressing certain thoughts aloud, which once said cannot be taken back. Also mentioned was an increased emotional pain and even physical pain that comes with resetting the self and starting afresh when they might have not been a sufficient reason for it, as noted James: *“You might create frustrations which needn’t be brought to the fore if somebody has actually sort of been successful and contented and satisfied and raised questions which actually could cause frustrations and difficulties in their lives that were not there in the first place”*.

In summary, many coaches agreed that what might initially seem beneficial for some coachees could potentially become a risk for others. They cited real-life examples where changes in self-perception led to irreversible changes such as ending relationships, leaving jobs and other decisions deemed too hasty, in retrospect.

### 4.3 Theme 3 - coach as instrument

#### 4.3.1 Coaches’ own identity

The significance of coaches’ own identities emerged more prominently than expected, unfolding naturally during interviews focused on the influence of a coach’s identity on the coaching process and outcomes. Coaches varied in their views of their professional identities. While some recognized a distinct coaching persona, others needed more time to think deeply about what this means. One coach reflected: *“If my identity as a coach is strongly fused with the results that I achieve, that’s going to significantly influence how I coach and is going to mean that my coaching is more driven around what I need as a coach to get my sense of identity as opposed to what the client really needs”*.

This observation revealed that coaches often instinctively use their personal identity stories when discussing identity’s role in coaching. This self-referencing was significant providing a framework that shaped their coaching methods and influenced their responses. Coaches with a psychodynamic approach were particularly aware of how their personal identities blend into their coaching practices. Generally, it became clear that a coach’s values, life experiences and aspirations influenced what they noticed, did not notice or sought out in their coaching with Ann, reflecting on this in her own practice and realizing her strong pull toward her identity, poignantly asking, *“Who is in the driving seat”*?

Overall, there was a notable difference in the level of this awareness among coaches, underscoring the diverse impacts of personal identity on their professional roles. Rachel, a therapeutically-aware coach articulated the deep connection between her personal identity and her professional approach: *“My approach to coaching and myself are inextricably linked. My approach to coaching is part and parcel of who I am.”* Margo self-referenced when reflecting on her comfortableness when coachees open up about personal battles: *“I love it when they do because it’s my comfort zone because I’ve had to navigate it for 45 years”.* Ann, reflecting on her personal commitment to authenticity, questioned how it might shape her coaching interactions, particularly when exploring the varied identities of her clients: *“I’ve got thing about authenticity so when I’m talking to my client (…), am I making that a thing because it’s important to me”*?

The discussion around coach identity unearthed significant themes of projection and transference. Some coaches highlighted the risk of excessive empathizing and mirroring, which could compromise the coaching environment. Others recognized the potential existence of themes that could trigger them adversely as coaches if they had unresolved issues themselves. The importance of recognizing both conscious and unconscious biases was also emphasized, especially when dealing with the intricate layers of clients’ identities.

In summary, reflecting on their roles within the coaching dynamic, there was a distinction in how coaches perceived their own selves as tools within the coaching process. Those with a background in therapeutic or psychological training were more adept at utilizing their self-awareness to navigate and leverage discomfort during sessions without succumbing to rescuer tendencies or other inadvertent forms of identification. For these professionals, the self was an invaluable tool that provided insights for both coach and their coachees. In contrast, those unable to retain boundaries reported feeling overwhelmed and energetically depleted, with some sessions even hinting at self-sacrifice.

#### 4.3.2 Coaches’ way of being

All coaches agreed on the essential role of rapport in their practice, particularly in identity coaching that required trust and connection during deeply personal explorations of self. A prevalent theme among the coaches was self-acceptance and compassion toward oneself and others. Coaches often discussed identity, life stories, meaning, purpose, awakening, personal truth, and a profound connection with the world in ways that highlighted their immense compassion. Describing their work, especially in relation to their clients’ sense of self or essence, terms they used interchangeably, their expressions softened, their pace slowed, and they radiated a genuine warmth and understanding toward their clients, irrespective of the individual stories. Compassion was not just a characteristic trait but intrinsically linked to how coaches perceived and conducted their leadership and executive development roles. Saul summed up the significant impact of this approach, saying: *“The ability to have self-acceptance and self-compassion through exploring identity I think contributes to, I would say 80% of a client’s progress in a coaching program”*.

Reflecting further on the attributes displayed during the coaching of identity, was a sense calmness, balance, curiosity and humility. All coaches agreed that the way of being was fundamental to successful identity coaching. As one coach recalled: *“You have to have enough of a connection with the people where you have to create enough safety in the room that people are open and honest but in a respectful way and compassionate way for it to be a very generative experience rather than a distressingly experience for people”*.

This blend of professional warmth, deep understanding, and emotional intelligence underscores the nuanced and impactful nature of coaching at the level of personal identity.

### 4.4 Theme 4 - methodology and tools

#### 4.4.1 Psychological safety

The exploration into how coaches work with themes related to identity produced a crucial theme of psychological safety. While psychological safety can be created by the coach’s way of being, coaches were more explicit in what they did to create this. All coaches agreed that ethically and effectively facilitating identity coaching necessitates the client’s preparedness to engage at such a profound level. They cited effective signposting, which proves challenging without a clear framework, as essential for readiness, as well as transparency about what is being discussed and depths of exploration, as noted by Tom referring to the coaching process: *“The minute it’s not transparent and it’s not clear to the client what the hell you are doing as a coach starts to impact on trust, especially in this domain because this goes deeper than beliefs and assumptions, does not it”*?

Psychological safety was also created using appropriate silence to allow thinking time. Coaches also reporting frequent re-contracting, involving check-in questions to evaluate how the was feeling and whether they wished to continue discussing the current topic. Coaches also strived to demonstrate their genuine compassion toward their coachees to help create a safe space. There was some disagreement whether coaches sharing their own identity stories served the interests of clients. While some viewed personal disclosure of how, for example, they achieved self-acceptance in context similar to coachee’s challenge, others saw it as inappropriate.

Some coaches have also recognized that it was important to give coachees time to reflect on some of the questions or themes in identity coaching to avoid the risk of the coachees *“feeling like they are not giving you what you need”* within the session (Lazarus, 2019).

Additionally, psychological safety was achieved through what the researcher described as “artful withdrawal from depth.” This approach acknowledges the deep psychological roots of identity issues and the necessity for coaches to remain aware of the boundaries of their skills and the session’s scope, with coaches reflexivity and skill of returning to a safe level of coaching exploration. It is crucial to add that such withdrawal, as explained by coaches, was conducted transparently, ensuring the coachees understood the reasons for stepping back, whether related to the depth of the issue or session practicalities. This method allowed for a safe and supportive withdrawal, empowering the coachees to feel in control and well-supported throughout the process.

#### 4.4.2 The “how to guide” of identity coaching

Given the variance in the coaches’ background, education and interest, the research provides examples of coaching methods deployed by the research participants. Even though some coaches were surprised at their own unconscious processes when working at the level of identity, they were able to articulate the “how” of identity coaching, as understood during interviews. It’s important to note, however, that all agreed that tools and techniques, while important, were secondary to the coach’s way of being.

Common techniques included a careful use of language, questioning techniques and strategic use of pauses. More specific approaches and exercises included: strength, values and beliefs-based activities, vision boards, 360-feedback surveys, perceptual position exercises, timeline exploration, psychometric tests, transactional analysis four-part identity framework, adult development-based identity framework, preparatory reading, reflective practices post-sessions, journaling, spatial activities, writing letters to one’s younger or older self, daily gratitude exercises, meditation, stakeholder mapping, storytelling, creative drawing of personal narration. Some coaches also employed innovative exercises designed to disrupt neurological patterns, alongside breathing techniques, walking sessions, and other dynamic activities.

Coaches’ ability to detect emerging identity issues within a session varied significantly. The notion of “depth” became a crucial scale. Coaches attentively assessed subtle cues such as sensory acuteness to changes in the coachee’s emotional state or the session’s energy dynamics, discomfort experienced by either party, despondent silences, emotional outbursts, or noticeable emotional reactions to coaching in general. Language cues were particularly telling, with phrases like *“I am not comfortable there,” “I’m stuck,” “I should,” “I do not know how I got here,” “I’m at a crossroads,” “I do not know who I am supposed to be,” “they do not understand me,” “they expect me”* and others providing insights into the coachee’s internal conflicts and identity crises. Phrases indicating habitual behaviors such as “typically,” “never,” “predictably” were also scrutinized to understand the pervasiveness and depth of the issues discussed, assessing how deeply these were embedded in the coachee’s psyche. For instance, one coach noted how a coachee’s team conflict reflected broader issues permeating other aspects of his life, illustrating the interconnectedness of personal and professional challenges.

### 4.5 Theme 5 - coach education

Most coaches reflected that they lack formal training in terms of coaching at the identity level and they observed that there was little to no explicit focus on this aspect of coaching during coaching qualifications. In a candid moment during the interview, Julianne joked: *“How can I be in coaching all this time and not really thought about identity lens much? Because I think about it a lot, I’m obsessed about my own identity, aren’t we all, particularly when you are a coach”*?

When prompted to identify which aspects of their coaching education were particularly beneficial for identity coaching, responses varied widely. Some highlighted their foundational understanding of values and beliefs, while others pointed to personal life experiences and heightened self-awareness as crucial learning points. Specific training approaches, such as psychodynamic methods and advanced Neuro-Linguistic Programming (NLP), which explicitly addressed identity themes, were recognized as useful yet only partially covering this expansive topic. Most coaches acknowledged that their most insightful learning occurred through continuous professional development (CPD), supervision, courses, and independent study, rather than through accredited coaching programs.

Looking ahead, many coaches expressed interest in expanding their knowledge of psychology and coaching psychology to better understand behavioral patterns and assist clients effectively in reevaluating self-perceptions linked to past experiences. Wanting to understand how the latest advances in neuroscience influence coaching practices and insights into identity management was also mentioned.

The consensus among interviewees was clear: it is essential for coaches to thoroughly understand the constructs of identity and the theories of identity formation. This knowledge should be applied creatively and expansively to avoid the pitfalls of a narrow and subjective approach to identity coaching.

Furthermore, all asserted that coach training that emphasizes creating an understanding of coaches’ own identity, both personal and professional would provide fundamental grounding and coaches’ self-awareness required to coach on issues related to identity safely and effectively. Supervision was also included and deemed crucial in advancing the coaches’ ability to coach at the level of identity.

## 5 Discussion

Given the recognition that periods of change often heighten individuals’ focus on identity (Woodward, 2004; Black et al., 2017), it is essential to examine the role of identity in coaching. As the coaching profession has expanded (Bresser and Wilson, 2010), it is increasingly seen as a tool to navigate the complexities of modern life (Stelter, 2013; Shoukry and Cox, 2018), offering significant societal benefits by fostering positive shifts in self-perception and attitudes. However, questions persist about its efficacy in addressing contemporary challenges in a rapidly evolving, fragmented world where individuals are constrained by, yet also active constructors of their identities (Smith et al., 2017). These individuals continually face internal conflicts, juggling stability with change, professional duties with personal identities, and current selves with potential future selves (Ibarra and Barbulescu, 2010). Grant (2017) points out that coaching often struggles to yield enduring change, which has sparked discussions that its true value today predominantly resides in the developmental sphere on advancing the individual’s being rather than just their doing (Bachkirova and Kauffman, 2009).

The interest in identity in coaching has also arisen from findings that identity plays a crucial role in Human Resource Development (Egan and Hamlin, 2014) and interventions aimed at shaping identity in this context prove highly effective in boosting individual performance and growth (Black et al., 2017), suggesting identity management and adaptability as more impactful than mere technical skills (Hall et al., 1997).

Considering this, the paper aimed to examine coaches’ knowledge, awareness, and understanding of identity issues and their choice of methods for addressing such topics in their coaching practice.

To advance this exploration, a thorough review of the literature was conducted to identify core themes and insights regarding identity. This review was crucial to present identity as a complex, nuanced and contradictory in nature, and with significant link to people’s sense of agency, adaptability, behaviors and decisions. Although the literature review confirmed that coaching is evolving toward an emphasis on addressing identity as a broad theme, it also highlighted significant gaps in the current body of knowledge, pointing out the need for more comprehensive resources to better equip coaches to engage with identity issues in their coaching practice. The literature has also offered very scant information on identity definition in coaching, as well as coaches’ understanding of this construct and practical implications of it on their work.

To address this, the researcher conducted 14 semi-structured interviews with executive coaches who provided vital insight on identity-related issues in coaching. The findings revealed that majority of coaches were indeed shifting toward a holistic, whole-person approach, with many already actively embracing it. Many recognized they had a preference to facilitate exploration and the development of a coherent sense of self and agency, as essential for a meaningful future (Whitmore, 2010; Bachkirova, 2012; Stelter, 2013; Stelter, 2018). This shift reflects a broad recognition among coaches of the importance of addressing the cognitive, emotional, behavioral, physiological, and sometimes even spiritual dimensions of their clients. As coaches increasingly helped their clients address challenges related to confidence, self-esteem or energy depletion, the coaches way of being demonstrating compassion, psychological safety and self-acceptance have emerged as critical attributes for effective identity coaching. This evolution in coaching practices marks a significant departure from traditional goal-oriented approaches, moving toward a more empathetic, humanistic perspective that prioritizes the coachee’s overall well-being over mere goal achievement. This approach aligns with broader societal concerns about human coping mechanisms and burnout, as highlighted by Han (2015) and Stelter (2018), who discuss the perils of ‘achievement compulsion’ and pervasive self-criticism. The shift toward compassionate, identity-focused coaching is seen as a positive development in the field, addressing deeper transformational needs rather than just behavioral adjustments.

The study also emphasized that to handle identity-related issues effectively requires coaches to possess sophisticated skills and a deeper understanding of coaching psychology. This knowledge is essential for coaches to identify and address unproductive cognitive and emotional patterns that influence their clients’ actions and reactions, and how to unpack these in a safe and self-sustaining way. Despite this need, many coaches reported a lack of training in dealing with identity issues. Furthermore, in the absence of a comprehensive theoretical framework on identity and readily accessible resources, some added that their preparation did not include a thorough examination of what identity entails or an assessment of potential risks before engaging in coaching at the identity level. All coaches agreed that a lack of awareness about their own identity can be a limiting factor in in creating effective coaching environments focused on identity work.

Additionally, with increasing focus on mental health in the workplace and organizations working to destigmatize issues related to human coping, clients are becoming more receptive to discussions that might have previously been considered too therapeutic for traditional coaching settings. The research recognizes that while coaching remains distinct from therapy, it increasingly offers a therapeutic benefit, helping coachees ready to engage deeply with their identities.

As the study advanced, it pinpointed key factors in identity coaching that could either support or impede the method’s effectiveness, and prompted a reassessment of its suitability given potential risks. These issues primarily pertained to the coaches’ personal identities and how these influenced their coaching approach. Educational background also played a crucial role. Most coaches embraced an “inside out” rather than “outside in” coaching methodology, focusing directly on the coachee’s identity. However, the study revealed a lack of consensus on a definitive identity definition among coaches, raising concerns about the potential overuse of this approach when it might not suit the coachee’s needs. Moreover, it was evident that coaches often interpreted identity through a subjective lens, influencing their coaching style, except for two coaches who reported significant benefits from using established frameworks to guide their sessions.

The concept of identity, as defined by Breakwell (2015), p. 10, is a blend of social psychological experiences and broader societal interactions. This definition underscores the complex psychological aspects of identity, suggesting that coaches who engage in this level of work should have a robust and nuanced understanding of identity, its formation, and its dynamic impact on an individual’s adaptability, agency, and resilience. This is particularly pertinent given the prevalent challenges in today’s executive coaching landscape, such as loss of control, workplace conflicts, directionlessness, overwhelm, mid-life crises, and anxiety. These elements underscore the necessity for coaches to possess a deep comprehension of identity to effectively navigate and mitigate such issues in their coaching practices.

The findings of this research paper have uncovered a notable gap in both the theoretical framework and established best practices for coaching at the identity level. It has also confirmed coaches’ keen interest in this area, alongside a recognition of the complex psychological dynamics involved. It is therefore strongly recommended that coaches and coach training providers develop a clearer understanding of the psychological factors and dynamics that manifest during coaching sessions. These insights are crucial as they address the intrapsychic pressures that often underlie the development of unproductive behaviors which some coaches may not be adept at working with, a concern highlighted by Berglas (2002). Furthermore, echoing Izod and Whittle (2013), there’s a strong case for coach training programs to include robust education on identity concepts and effective methods by which coaches can enhance their self-awareness. Such training helps coaches navigate the inherent unpredictability, paradoxes, and risks without becoming entrenched in their fixed identities, instead using their identities as useful frameworks for interpretation across various contexts. Additionally, this research underscores a desire among coaches to amalgamate diverse approaches and methodologies into a single, easily accessible resource to better navigate the complexities of identity within their practice.

As executive coaching increasingly embraces a holistic, whole-person approach, there’s also a recommendation to foster dialog among practitioners, academic bodies, and organizations that employ coaching services. This dialog should explore both the potential benefits and the limitations of identity coaching, aiming to refine the approach and position it as a powerful tool for transformative self-reflection and personal growth.

Looking ahead, the initial focus for future research should be on gaining a deeper understanding of how coachees experience coaching at the identity level. This should be followed by an in-depth study into the effectiveness of identity-based coaching approaches, assessing their impact and refining the methodology based on empirical evidence.

## Data Availability

The original contributions presented in the study are included in the article/Supplementary material, further inquiries can be directed to the corresponding author.
