# Supplementary material for: Exploring identity in coaching – insights into coaches’ understanding and approach
Source: Front Psychol. 2025 Feb 5;15:1445643. doi: 10.3389/fpsyg.2024.1445643 (PMC11835513; doi:10.3389/fpsyg.2024.1445643)
Supplement: Supplementary file 1 [file Data_Sheet_1.PDF]

Appendix 1: Interview Guiding Questions

Interview questions were aligned with the two aforementioned areas of exploration. While the researcher listened out to any unanticipated insights, the questions were designed to serve as prompts as w when the interviewer felt that the interviewee was veering off the subject.

1. **Coaches' knowledge, awareness and understanding of the subject of identity in the process of coaching**

   1.1. Tell me about yourself as a coach and your coaching philosophy or approach to coaching?
   1.2. What kinds of issues, in general, do clients typically bring to the sessions?
   1.3. My focus is on understanding how we, as coaches, deal with issues concerning clients' identity, which may present itself explicitly or disguised under other terms, words or terms. So, before we explore that, I would like to ask you – how do you define identity?
   1.4. How does identity present itself in coaching?
   1.5. How do you recognise that identity-related theme surfaces in the coaching session?
   1.6. Is there anything else to that you might consider 'oh, this is identity related issue'?
   1.7. As far as clients are concerned – what are the benefits and the risks to the client when and the risks in bringing issues / themes concerning identity to coaching?
   1.8. How comfortable are you in discussing issues related to client's identity?
   1.9. Can you think of a challenging coaching situation - what made it a challenge?
   1.10. What is the impact of a person's sense of identity on them?
   1.11. Could there be any incompatibility between coaching and working with themes related to client's identity?

2. **Coaches' choice of methodology and tools when coaching on issues of identity**

   2.1. Do you use the word 'identity' in your coaching practice? What words do you use?
   2.2. What approach do you take when coaching on issues or themes related to client's identity?
   2.3. What tools or frameworks do you deploy when coaching clients at identity level?
   2.4. How do you decide what approach works best?
   2.5. Which part of your coach education specifically enabled you to address client's identity topics?
   2.6. What specific training do you think might help coaches to work with issues related to identity?
   2.7. What can be done to supported coaches be more effective at working with clients when issues of identity present themselves?
   2.8. What else is there for you to learn in the area of coaching around issues of identity?
